# A Thirty-seven-Pound Tumor of the Left Supraseual Capsule

**Published:** 1883-01

**Authors:** 


### Original Translations and Abstracts.

A Thirty-seven-Pound Tumor of the Left Supraseual Capsule—Extirpation—Recovery.—Dr. Bruntzel has added another to the small list of successful extirpations of the kidney. The patient, a woman aged 35, had noticed that a tumor had been slowly increasing in size from the time it first was manifest, in the year 1877. No pain accompanied its development until it began to press upon the adjoining nerves and viscera. Disturbances of nutrition, the pains in the extremities and the mechanical obstruction it offered to movements of the simplest kind induced the patient to submit to operation, in spite of Dr. Bruntzel's statements that death might occur during, and was liable to follow, the operation. Percussion revealed the outline of the tumor clearly; it reached from the xiphoid cartilage to the symphysis, along which line the incision was made. The tumor was seen to be retroperitoneal; but its origin could not be made out, so far did it extend in all directions. During the removal, when numerous large vessels had to be tied, the kidney was found deeply imbedded in the centre of the mass, and was removed with the growth.

The patient, all this time under the influence of combined morphia-chloral-chloroform narcosis, now showed alarming symptoms. Artificial respiration and hypodermics of ether were successfully resorted to, and after washing out the cavity, which was so large that at every respiration a part of the stomach was sucked down into it, the wound was closed, two drainage tubes being left in. All went well until on removing one of the sutures ten days after the operation, sudden collapse followed, and lasted three hours. This was caused by a small rupture of the intestine, for fecal matter exuded from the opening where the drainage tubes were placed. This was caused either from necrosis due to puncture or compression of a vessel by the suture, or from injury to the intestine in putting in the sutures. The fistula soon closed and the patient recovered.

The tumor was a fibroma, thirty-seven and one-half pounds in weight, made up of numerous lobulated masses varying in size from that of an orange to that of a man's head, and divided from one another by slight depressions. A yellow spot of commencing degeneration was found at the centre, and the vessels about it were the seat of thrombi. Imbedded in the tumor was the kidney, whose *structure was wholly normal.*—[*Berliner Klinische Wochenschrift, Dec.* 4, 1882.

